# Exclusive Maternal Milk Compared With Exclusive Formula on Growth and Health Outcomes in Very-Low-Birthweight Preterm Infants: Phase II of the Pre-B Project and an Evidence Analysis Center Systematic Review

**DOI:** 10.3389/fped.2021.793311

**Published:** 2022-02-25

**Authors:** Sarah N. Taylor, Tanis R. Fenton, Sharon Groh-Wargo, Kathleen Gura, Camilia R. Martin, Ian J. Griffin, Mary Rozga, Lisa Moloney

**Affiliations:** ^1^Division of Neonatology, Department of Pediatrics, Yale School of Medicine, New Haven, CT, United States; ^2^Community Health Sciences, Institute of Public Health, Alberta Children's Hospital Research Institute, Cumming School of Medicine, University of Calgary, Calgary, AB, Canada; ^3^Nutrition Services, Alberta Health Services, Calgary, AB, Canada; ^4^Nutrition Services, Alberta Health Services, Calgary, AB, Canada; ^5^Departments of Nutrition and Pediatrics, Case Western Reserve University at MetroHealth Medical Center, Cleveland, OH, United States; ^6^Clinical Research Program, Department of Pharmacy, Boston Children's Hospital, Boston, MA, United States; ^7^Division of Translational Research, Department of Neonatology, Harvard Medical School, Neonatal Intensive Care Unit (NICU), Beth Israel Deaconess Medical Center, Boston, MA, United States; ^8^Biomedical Research Institute of New Jersey, Cedar Knolls, NJ, United States; ^9^Department of Pediatrics, Morristown Medical Center, Morristown, NJ, United States; ^10^Academy of Nutrition and Dietetics, Evidence Analysis Center, Chicago, IL, United States; ^11^Academy of Nutrition and Dietetics, Evidence Analysis Center, Chicago, IL, United States

**Keywords:** mother's milk, maternal milk, preterm infant, very low birthweight, enteral nutrition, systematic review

## Abstract

As part of the Pre-B Project, a systematic review was conducted to evaluate associations between exclusive maternal milk (≥75%) intake and exclusive formula intake and growth and health outcomes in very-low-birthweight (VLBW) preterm infants. The protocols from the Academy of Nutrition and Dietetics' Evidence Analysis Center and the Preferred Reporting Items for Systematic Reviews and Meta-Analyses (PRISMA) checklist were followed. Thirteen observational studies were included; 11 studies reported data that could be synthesized in a pooled analysis. The evidence is very uncertain (very low quality) about the effect of exclusive maternal milk on all outcomes due to observational study designs and risk of selection, performance, detection, and reporting bias in most of the included studies. Very-low-quality evidence suggested that providing VLBW preterm infants with exclusive maternal milk was not associated with mortality, risk of necrotizing enterocolitis, sepsis, or developing bronchopulmonary dysplasia, as compared with exclusive preterm formula, but exclusive maternal milk was associated with a lower risk of retinopathy of prematurity (very low certainty). Results may change when additional studies are conducted. There was no difference in weight, length, and head circumference gain between infants fed fortified exclusive maternal milk and infants receiving exclusive preterm formula; however, weight and length gain were lower in infants fed non-fortified exclusive maternal milk. Given the observational nature of human milk research, cause-and-effect evidence was lacking for VLBW preterm infants.

**Systematic Review Registration:**

https://www.crd.york.ac.uk/prospero/display_record.php?RecordID=86829, PROSPERO ID: CRD42018086829.

## Introduction

Human milk contains nutritional and immunologic factors that have been associated with healthy development in full-term infants. In a meta-analysis of cohort and cross-sectional studies, breastfeeding has been associated with decreased risk of infection, autoimmune diseases, and cancer in full-term newborns (1). For infants born preterm (<37 weeks), human milk feeding has demonstrated associated benefits including fewer infections and fewer inflammatory diseases such as necrotizing enterocolitis (NEC) compared with formula feeding (2–5). For very-low-birthweight (VLBW) preterm infants (≤1,500 g), therefore, human milk fortification is recommended at least during the initial hospitalization (6). Compared with full-term infants, VLBW infants are at higher risk for nutritional deficiencies and diseases such as NEC. According to the analysis conducted in 2008, ~7% of VLBW infants develop NEC. Systematic reviews (SRs) that include infants with higher birthweights increase the heterogeneity and indirectness of the evidence. Populations with a birthweight of <1,500 kg should lower heterogeneity, increase directness, and provide results about the subpopulation that require more neonatal intensive care.

SRs have been conducted on the outcomes after human milk intake in VLBW infants (7, 8). Miller et al. conducted an SR in 2018 to evaluate the association between human milk feeding and morbidity, and Saguna conducted an SR in 2021 on the association between human milk feeding and short-term growth in VLBW preterm infants (7, 8). Both SRs compared the intake of exclusive human milk with exclusive formula, any milk compared with formula, and pasteurized compared with unpasteurized human milk, as well as dose–response associations with human milk intake. The authors of these two SRs included maternal milk (MM) and donor milk studies, and a sub-analysis was not provided for MM. MM has unique advantages because it is tailored to each parent–infant dyad. Donor milk has been explored as a comparable alternative to MM; however, the biochemical profiles of MM differ from those of donor breast milk (9). To improve the directness of the evidence, it is imperative to restrict to articles that report milk sources, as well as quantity or proportion of intake, when evaluating the association between MM and outcomes.

The limitations of studies examining the effects of human milk are substantial. Due to the maternal right to choose whether a mother provides her milk and the high prevalence of lactation insufficiency, infants cannot be randomized to MM vs. infant formula. Additionally, in these observational studies, social determinants of health are associated with the maternal choice to provide milk, with maternal factors related to lactation insufficiency and with health outcomes. Thus, the specific benefit of MM may be difficult to differentiate from other factors known to influence health outcomes.

Therefore, under Phase II of the Pre-B Project, the Evidence Analysis Center (EAC) Preterm Panel undertook SRs to develop evidence-based nutrition recommendations for VLBW infants and a foundation upon which to build future studies. The Panel conducted several SRs to support human milk recommendations including MM compared with formula, MM dose–response, fortification of MM with donor milk compared with formula, and donor milk compared with formula (10). The objective of this supporting SR was to examine the research question: in VLBW (≤1,500 g at birth) preterm infants, what is the association between exclusive MM (≥75%) and exclusive formula intake on growth and health outcomes?

## Methods

This SR followed the protocols from the Academy of Nutrition and Dietetics' EAC (11) and adhered to the parameters described on the Preferred Reporting Items for Systematic Reviews and Meta-Analyses (PRISMA) checklist (12). This SR was part of the larger Pre-B Project to inform an evidence-based practice guideline on enteral nutrition for VLBW preterm infants (13) and was prospectively registered at PROSPERO (ID CRD42018086829) (14). For the purposes of this review, the term “maternal milk” is used. The Preterm Panel recognizes that not all people who give birth and are lactating identify as women, but since early postpartum milk has a different composition from mature milk, the milk provided by the biological mother of the infant is referred to as “maternal milk.”

### Eligibility Criteria

The research question was formulated according to the Population, Intervention/Exposure, Comparison, Outcome (PICO) format. To be included, studies were required to address each part of the PICO question. The target population was preterm infants weighing ≤1,500 g at birth. Studies were excluded if authors did not limit inclusion to infants ≤1,500 g at birth or if reported mean birthweight plus two standard deviations suggested that infants with birthweight >1,500 g had been included. To be included, studies must have compared infants receiving ≥75% of intake from MM with infants receiving exclusive formula. The authors defined exclusive MM intake as 75%, as that is the percentage commonly reported in preterm infant feeding literature. The outcomes of interest were defined *a priori* and included health and growth outcomes, including mortality, NEC, sepsis, bronchopulmonary dysplasia (BPD), retinopathy of prematurity (ROP), visual acuity, bone mineralization, weight and length gain, body composition, and head circumference.

Studies taking place in countries without developed economies according to the United Nations classification were excluded because neonatal intensive care unit (NICU) and feeding practices may vary considerably compared with those in countries with developed economies. Included studies were limited to those published in the English language due to resource constraints. Articles published after the *a priori* specified date of January 1, 1980, until the final search date of June 2020 were eligible for inclusion. A full description of the eligibility criteria can be found in Table 1.

**Table 1:** Inclusion criteria for the SR examining the association between MM intake and formula intake on growth and health outcomes in VLBW preterm infants.

**Criteria**	**Included**	**Excluded**
Peer-review status	Published in a peer-reviewed journal	Non-peer-reviewed articles, such as government reports and gray literature
Population	Preterm infants ≤ 1,500 g	Term infants or infants >1,500 g
Location	Countries with developed economies according to United Nations (15)	Countries with developing economies
Search dates	January 1, 1980, to November 1, 2018: Embase, PubMed, CINAHL Complete, Cochrane central register of controlled trials, and Cochrane Database of Systematic Reviews databases. November 1, 2018, to June 5, 2020 (PubMed only)	Outside inclusion dates
Exposure	≥75% of intake from MM	Intake of MM <75% or not reported; donor milk
Comparison	Exclusively formula fed	Exclusively formula fed
Study design	Cohort studies, randomized or clinical trials	All other study designs
Outcomes	**Mortality/survival morbidity** [e.g., retinopathy of prematurity (ROP), sepsis, bronchopulmonary dysplasia (BPD), rickets, allergies, anemia, and necrotizing enterocolitis (NEC)] **Growth** [weight/length/head circumference change (cm/week) including growth velocity: change in *z*-scores, g/kg/day] **Anthropometrics** (weight/BMI, length, head circumference including *z*-scores, body composition) **Development** (neuro/cognitive, motor, vision/retinal, behavior) **Bone mineralization** **Gastrointestinal health** (days on TPN, or time to full enteral feeds) **Adverse events/safety** (tolerance including metabolic acidosis, adverse events)	Other outcomes not indicated in inclusion criteria
Language	Articles published in the English Language	Articles not published in the English language

*SR, systematic review; MM, maternal milk; VLBW, very low birthweight; BMI, body mass index; TPN, total parenteral nutrition*.

### Search Plan

A literature search was conducted using Embase, PubMed, CINAHL Complete, Cochrane central register of controlled trials, and Cochrane Database of Systematic Reviews databases. Our primary search was conducted as part of a greater SR supporting an evidence-based practice guideline on enteral nutrition in preterm infants (13) and was updated during guideline development, with the most recent search conducted on June 5, 2020, using the PubMed database. Terms of interest included the following: preterm, very low birthweight, 1,500 grams, infant, mothers' milk, MM, breastfed, formula, human milk, expressed milk, and enteral. Relevant SRs were hand-searched for potentially qualifying primary research studies that may have been missed in the database searches.

### Study Selection

Each title/abstract was screened independently by at least two experienced practitioners from the Preterm Panel or EAC SR methodologists using Abstrakr software (16). All included title/abstracts progressed to full-text review. Each study was reviewed for inclusion according to eligibility criteria by at least two Preterm Panel members. Conflicts during the title/abstract and full-text review phases were settled through consensus or discussion with the full Preterm Panel. Each stage of the study selection process was documented on a PRISMA flow diagram (12).

### Data Extraction

Data from included articles were extracted by trained Evidence Analysts onto a standardized data extraction template (11) and were reviewed for accuracy by the lead analyst (MR) or project manager (LM). Extracted data included the following: bibliographic information; eligibility criteria; study location and funding source; sample size; participant characteristics (birthweight, gestational age, race, sociodemographic data, and comorbidities); proportion or quantity of total intake from MM or formula, types of enrichment, fortification, and infant formula when applicable; and results of outcomes that were prioritized *a priori*.

### Risk of Bias and Quality of Evidence

For each included study, risk of bias was assessed independently by an Evidence Analyst and a Lead Analyst or Project Manager using the Academy of Nutrition and Dietetics' Quality Criteria Checklist (17). This tool uses guiding sub-questions to determine risk of selection, attrition, performance, detection, and reporting bias. Discrepancies were resolved by a third reviewer.

The quality of evidence for each outcome was determined using the Academy (17) and GRADE (18) methods and GRADE recommended terminology (19). The outcomes were graded according to the study design, risk of bias of the included studies, sample sizes of studies reporting the outcome, consistency in findings between studies, generalizability, precision, effect size, and other factors. Risk of bias and quality of evidence determinations were reviewed by the Preterm Panel. Quality/certainty of the evidence was rated as high, moderate, low, and very low.

### Synthesis of Results

All included studies were described in a study characteristics table and summarized narratively by the outcome. Certainty/quality of evidence was summarized by the outcome in a GRADE summary of findings table. If more than one study included quantitative results that could be pooled, they were included in a meta-analysis using a random-effects model. Studies reporting sample size and mean effect size with variance for continuous variables, or event numbers for categorical variables, for each group were included in the meta-analyses. Studies that did not report data that could be pooled in a meta-analysis were described in narrative synthesis only. Continuous variables were summarized using mean difference (MD) or standardized MD (SMD) between groups with 95% CI. Categorical variables were described as odds ratio (OR) (95% CI). Meta-analyses were performed using RStudio (20) and reported in forest plots. Publication bias was tested for using funnel plots, and heterogeneity was determined using *I*^2^ measures. The Preterm Panel composed a conclusion statement that directly answered the PICO question for each outcome based on the narrative and quantitative results and evidence quality/certainty.

## Results

**Figure 1:**
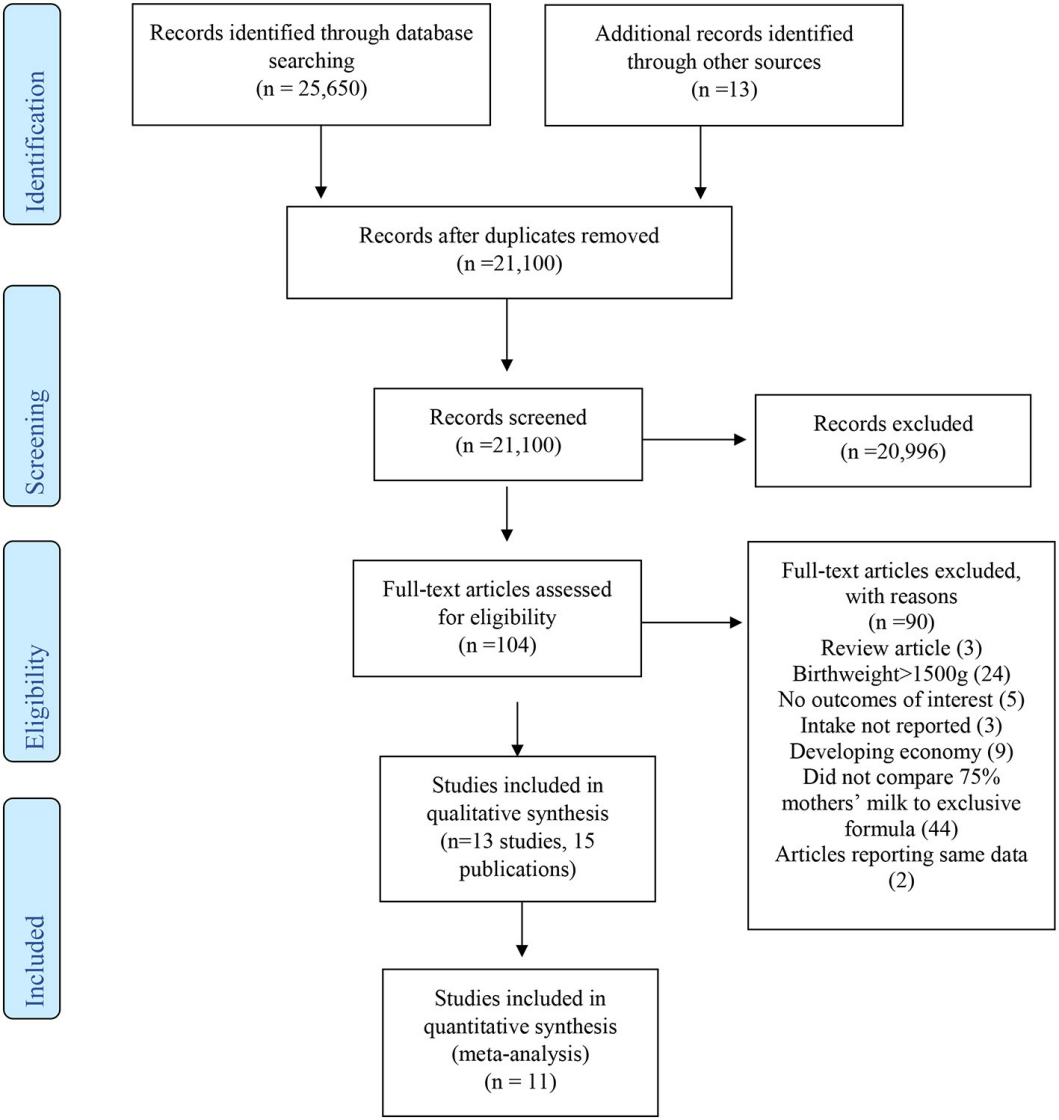
PRISMA flow diagram describing article inclusion for a systematic review examining the question: In VLBW preterm infants (≤1,500 g at birth), what is the association between ≥75% MM vs. exclusive formula intake on growth and health outcomes?

A total of 21,066 unique studies were identified in the databases and hand-searches for the entire Pre-B preterm nutrition guideline project. For the current SR, 104 full-text articles were reviewed, and 13 studies (represented in 15 articles) (21–35) were included in narrative synthesis, with 11 studies reporting quantitative data that could be synthesized in pooled analysis (Figure 1, Table 2). Sample sizes ranged from 9 to 498 participants, and study duration ranged from 2 to 27 weeks. Study designs included 10 prospective cohort studies (21–23, 25–30, 32, 34, 35), 2 retrospective cohort studies (31, 33), and 1 non-randomized trial. The percentage of MM intake and fortification can be found in Table 2. To be included in the MM group, infants must have consumed at least 75% of the intake from MM. However, some study authors did not describe intake of the remainder of feedings, which may have been up to 25% of intake. The summary of findings for each outcome is described in Table 3.

**Table 2:** Study characteristics and results of articles evaluating the association between ≥75% MM and exclusive formula intake and growth and health outcomes in VLBW (≤1,500 g) preterm infants.

**Study (author, study design, PMID)**	**Sample characteristics**	**Intervention/duration**	**Results**		**Conclusions and confounding variables**	**Risk of bias domain(s)**
			**MM group (≥75% of intake)**	**Formula group**		
Mortality/survival						
Manzoni et al. (31) Retrospective cohort study, observational analysis of multicenter RCTs of other topics PMID 23809355	*Mean (±SD) birthweight (g)* MM: 1,125 (±247) Preterm formula: 1,100 (±272) % SGA: NR *Mean (±SD) GA* *(weeks)* MM: 29.4 (±2.5) Preterm formula: 29.2 (±2.8)	MM was provided exclusively, and fortification was not described Formula was provided exclusively and was “standard preterm formula” *Mean duration of NICU days* MM: 50 Preterm formula: 54	MM (*N* = 314) % death prior to discharge 3.6	Preterm formula (*N* = 184) % death prior to discharge 8.2	There was no statistically significant difference in death prior to discharge according to feeding group (*p* = 0.18) Results were not adjusted for confounding variables. SES and maternal smoking were not described.	Risk of selection and attrition bias.
Morbidities						
Hendrickse et al. (29) Prospective cohort study PMID 6510430	*Mean birth weight (g):* MM: 1,171 LBW formula: 1,214 *% SGA:* NR *Mean GA (weeks):* 30	Infants received >95% of intake from respective milk. MM was supplemented with SMA Goldcap in some cases. Comparison infants low birth weight (LBW) formula with 76 kcal, 1.8 g/protein, 100 mg calcium, and 50 mg phosphorus/100 ml formula Infants spent at least 4 weeks on study milk	MM (*N* = 32) N NEC 6 weeks: 3	LBW formula (*N* = 34) N NEC 6 weeks: 3	There was no difference in incidence of NEC at 6 weeks	Risk of selection, attrition, performance, detection, reporting bias
Manzoni et al. (31) Retrospective Cohort Study, observational analysis of	*Mean (±SD) birthweight (g)* MM: 1,125 (±247) Preterm formula: 1,100 (±272)	MM was provided exclusively, and fortification was not described Formula was provided	MM (*N* = 314) N (%) ROP (threshold) 4 (1.3) % NEC (≥2nd stage) 1.3	Preterm formula (*N* = 184) N (%) ROP (threshold) 22 (12.3) % NEC (≥2nd stage) 4.1	Infants receiving exclusive MM had an OR (95% CI) of 0.14 (0.12, 0.62) of all stages of ROP compared with those receiving preterm formula (*p* = 0.0004)	Risk of selection and attrition bias
Multicenter RCTs of other topics PMID 23809355	% SGA: NR *Mean (±SD) GA* *(weeks)* MM: 29.4 (±2.5) Preterm formula: 29.2 (±2.8)	Exclusively and was “standard preterm formula” *Mean duration of NICU days* MM: 50 Preterm formula: 54	% Late-onset sepsis 13.4	% Late-onset sepsis 17.3	There were no significant differences in odds of NEC or late-onset sepsis between groups at hospital discharge (not adjusted for confounders and no data provided)	
Mol et al. (34) Prospective Cohort Study PMID 29784603	*Mean (±SD) birthweight (g)* MM: 1,210 (±161) Preterm formula: 1,240 (±180) *% SGA: NR* *Median (IQR) GA (weeks)* MM: 29 (28–32) Preterm formula: 29 (28– 31.75)	Infants received exclusive MM (“standard” dose of fortifier (Bebilon HMF, Nutricia) beginning at 140 ml/kg/day) or exclusive preterm formula Outcomes measured at 40 weeks PMA	MM (*N* = 11) N (%) BPD 6 (54.5) N (%) NEC 2 (18.2) N (%) Sepsis 3 (27.3) N (%) ROP (requiring laser coagulation) 0 (0)	Preterm formula (*N* = 23) N (%) BPD 6 (26.1) N (%) NEC 4 (17.4) N (%) Sepsis 8 (34.8) N (%) ROP (requiring laser coagulation) 2 (8.7)	There were no significant differences in the number of infants with BPD, NEC, sepsis, or ROP (requiring laser coagulation) between groups at 40 weeks PMA Results were not adjusted for confounding variables	Risk of selection and performance bias
Anthropometrics						
Atkinson et al. (21)	*Mean Birthweight (g):* 970 All infants AGA	Infants were exclusively fed MM (fortification not	MM (*N* = 8)	SMA 20 (Balance 1) or 24 (Balance 2) (*N* = 8)	There were no differences in weight, length, or head	Risk of selection, performance,
Prospective cohort study (Balance study) PMID 7277107	GA: 28.3 weeks	mentioned) or formula (SMA20 or SMA24) Balance studies were conducted at the end of the 1st and 2nd weeks of the study	Weight Data only presented in figure. Length Data only presented in figure. Head circumference Data only presented in figure.	Weight Data only presented in figure. Length Data only presented in figure. Head circumference Data only presented in figure.	circumference between groups in either balance study (data in figure only) Results were not adjusted for confounding variables	detection bias
Atkinson et al. (22) Prospective cohort study (Balance studies) PMID 6848738	*Mean (±SEM) birthweight (g):* MM 1,060 (±77) Formula 1,065 (±55) All infants were AGA *Mean (±SEM) GA* *(weeks)* MM 28.6 (±0.7) Formula 28.4 (±0.7)	Fortification of MM was not described. Infants in the formula group received 67 kcal/dl formula, then 80 kcal/dl after 8 days postpartum Study duration was 28 days	MM (*N* = 5) Mean (±SE) weight change (g/day) 15 (±2) Mean (±SE) length change (cm/week) 1.0 (±0.1) Mean (±SE) head circumference change (cm/week) 1.0 (±0.1)	Formula (*N* = 5) Mean (±SE) weight change (g/day) 27 (±5) Mean (±SE) length change (cm/week) 1.2 (±0.1) Mean (±SE) head circumference change (cm/week) 1.3 (±0.1)	At 4 weeks, infants receiving 67 and 80 kcal/dl formula had a significantly greater increase in weight gain, head circumference (*p* < 0.01 for both), and length (*p* < 0.05) than the group receiving MM. This trend began at 1 week and persisted until 4 weeks Results were not adjusted for confounding variables	Risk of selection, attrition, performance bias
Chan et al. (26) Post-discharge Prospective Cohort study PMID 8355124	*Mean (±SEM) birthweight (g)* MM: 1,191 (±59) Standard formula: 1,215 (±78) LBW formula: 1,197 (±85) Premature formula: 1,128 (±48) % SGA: NR *Mean (±SEM) birth GA (weeks)* MM: 30 (±1) Standard formula: 29.0 (±0) LBW formula: 30 (±1) Premature formula: 29 (±0) *Mean (±SEM) age at start of intervention (days)* MM: 54 (±6) Standard formula: 51 (±7) Low birthweight formula: 54 (±8) Premature formula: 59 (±4)	Infants were outpatient MM was fortified during hospitalization Study formulas were 20 kcal/oz Intervention duration was 120 days	MM (*N* = 16) Mean (±SEM) weight (g) discharge: 2,037 (±61) 16 weeks: 4,620 (±178) Mean (±SEM) length gain (mm/day) 16 weeks: 1.02 (±0.04)	Standard formula (*N* = 15) LBW formula (N = 14) Premature formula (*N* = 14) Mean (±SEM) weight (g) Standard formula Discharge: 2,140 (±82) 16 weeks: 5,020 (±198) LBW formula Discharge: 2,114 (±57) 16 weeks: 5,288 (±199) Premature formula Discharge: 2,217 (±58) 16 weeks: 5,150 (±204) Mean (±SEM) length Gain (mm/day) Standard formula 16 weeks: 1.19 (±0.06) LBW formula 16 weeks: 1.21 (±0.05) Premature formula 16 weeks: 1.23 (±0.05)	Infants in each formula group were heavier than the infant's receiving MM at 16 weeks post-discharge (*p* < 0.01). Infants receiving LBW or premature formula had significantly greater length gain than infants receiving MM at 16 weeks post-discharge (*p* < 0.05) Results were not adjusted for confounding variables	Risk of selection, attrition, performance, detection bias
Doege et al. (27) Prospective cohort study PMID 17655982	*Mean (±SD) birthweight (g)* MM: 822 (±133) Preterm formula*: 832 (±132)* *% SGA: NR* *Mean GA (weeks):* *: 26.3 (±0.9)* Preterm formula*: 26.6* *(±0.8)* *Age at start of observation: 3 weeks postnatal*	Infants received either MM fortified with protein and phosphorus (≥80% of feedings) or exclusive preterm formula Observation duration: ~12 weeks	MM (*N* = 60) Mean (±SD) body weight (g) 3 weeks postnatal: 936 (±152) 38 weeks GA: 3,004 (±116) Mean (±SD) body length (cm) 3 weeks postnatal: 36 (±0.9) 38 weeks GA: 49 (±2) Mean (±SD) head circumference (cm) 3 weeks postnatal: 26 (±1.1) 38 weeks GA: 34 (±1.1)	Preterm formula (*N* = 60) Mean (±SD) body weight (g) 3 weeks postnatal: 940 (±146) 38 weeks GA: 3,010 (±170)	There were no differences in weight, length, or head circumference between groups at 38 weeks GA, but between-group changes were not reported	Risk of selection, attrition, performance bias
Genzel Boroviczeny et al. (28) Prospective Cohort Study PMID 9438148	*Mean (±SD) birthweight (g):* MM: *963 (±245)* *Formula: 829 (±159)* *% SGA: NR* *Mean (±SD) GA* *(weeks)* *MM 26 (±1.3)* *Formula: 27 (±1.3)*	Infants whose mothers did not breastfeed (fortification not described) received formula (50% MCT, 12% linoleic and 1% α-linolenic acid) Observed until 7 weeks of life	MM (*N* = 18) Mean (±) SD weight (g) Birthweight: 963 (±245) 7 weeks: 1,346 (±336)	Formula (*N* = 11) Birthweight: 829 (±159) 7 weeks: 1,243 (±293)	There was no difference in weight between groups at 7 weeks Results were not adjusted for confounding variables	Risk of selection, performance, detection bias
Hendrickse et al. (29) Prospective cohort study PMID 6510430	*Mean birth weight (g):* MM: 1,171 LBW formula: 1,214 *% SGA:* NR *Mean GA (weeks):* 30	Infants received >95% of intake from respective milk. MM was supplemented with SMA Goldcap in some cases. Comparison infants low birth weight (LBW) formula with 76 kcal, 1.8 g/protein, 100 mg calcium, and 50 mg phosphorus/100 ml formula Infants spent at least 4 weeks on study milk	MM (*N* = 10) Mean (±SE) weight gain (g/kg/week) Weeks 2–6: 102 (±4.8)	LBW formula (*N* = 14) Mean (±SE) weight gain (g/kg/week) Weeks 2–6: 124 (±5.9)	Weekly weight gain from weeks 2 to 6 was significantly higher in the LBW formula group compared with the MM group (*p* < 0.02) Results were not adjusted for confounding variables	Risk of selection, attrition, performance, detection, reporting bias
Modanlou et al. (32) Prospective cohort study PMID 3761107	*Mean (±SD) birthweight (g)* MM + fortifier: 1,086 (±161) Premature formula: 1,160 (±194) All infants were AGA	Infants in MM group received ≥90% MM; fortified to 24 kcal/fluid oz (per 100 ml MM: 14 kcal, 0.7 g protein, 2.7 g carb, vitamins, and minerals) Premature formula was 24 kcal/oz Intervention continued until discharge or until infant was ≥1,800 g. Average duration: 33.1 (±7.5) days	MM + fortifier (*N* = 8) Mean (±SD) weight gain (g/day) 29.4 (±3.7) Mean (±SD) head circumference growth (cm/week) 1.09 (±0.07)	Premature formula (*N* = 12) Mean (±SD) weight gain (g/day) 32.5 (±3.3) Mean (±SD) head circumference growth (cm/week) 1.16 (±0.19)	There were no differences in weight, length, or head circumference between groups at ~30 days Results were not adjusted for confounding variables	Risk of selection, performance bias
	*Mean (±SD) age at start of intervention (days):* MM + fortifier 9.8 (±3.8) Premature formula: 8.7 (±4.8)		Mean (±SD) length growth (cm/week) 0.99 (±0.40)	Mean (±SD) length growth (cm/week) 1.2 (±0.29)	Infants fed fortified MM and those fed high-calorie formula had greater weight gain, head circumference, and length compared with infants receiving unfortified MM	
	GA: NR					
Mol et al. (34) Prospective cohort study PMID 29784603	*Mean (±SD) birthweight (g)* MM: 1,210 (±161) Preterm formula: 1,240 (±180)	Infants received exclusive MM (fortified beginning at 140 ml/kg/day) or exclusive preterm formula	MM (*N* = 11) Mean (±SD) weight (g) Birth: 1,210 (±161) 40 weeks PMA: 3,336 (±385) Mean (±SD) length (cm) Birth: 40.3 (±3.0) 40 weeks PMA: 50 (±2)	Preterm formula (*N* = 23) Mean (±SD) weight (g) Birth: 1,240 (±180) 40 weeks PMA: 3,683 (±690) Mean (±SD) length (cm) Birth: 39.8 (±2.8) 40 weeks PMA: 52 (±3)	Infants receiving preterm formula were heavier (*p* = 0.02) and had greater head circumference (*p* = 0.002) than MM fed infants at 40 weeks PMA, but changes between groups were not compared	Risk of selection and performance bias
	% SGA: NR *Median (IQR) GA (weeks)* MM: 29 (28–32) Preterm formula: 29 (28– 31.75)		Mean (±SD) head circumference (cm) Birth: 27 (±1.7) 40 weeks PMA: 34.6 (±1.0) Mean (±SD) fat-free mass (FFM) (kg) 40 weeks PMA: 2.808 (±0.28) Mean (±SD) fat mass (FM) (kg) 40 weeks PMA: 0.529 (±0.11)	Mean (±SD) head circumference (cm) Birth: 26.9 (±1.7) 40 weeks PMA: 36.1 (±1.8) Mean (±SD) FFM (kg) 40 weeks PMA: 3.066 (±0.52) Mean (±SD) FM (kg) 40 weeks PMA: 0.617 (±0.18)	There was no difference in length, FM, or FFM between groups at 40 weeks PMA, but changes between groups were not compared, and there were no baseline values reported for FM and FFM Results were not adjusted for confounding variables	
Morlacchi et al. (33) Retrospective cohort study PMID 29529139	*Mean (±SD) birthweight (g)* MM: 1,214.8 (±246)	Infants received exclusive MM or preterm formula from birth to discharge.	MM (*N* = 17) Mean (±SD) weight change from birth to discharge (z-score)	Preterm formula (*N* = 15) Mean (±SD) weight change from birth to discharge (z-score) −0.6 (±0.7) Mean (±SD) Length Change from birth to discharge (z-score)	There was significantly more decrease in weight z-score from birth to discharge in the MM group	Risk of selection, performance, detection, reporting bias
	Preterm formula: 1,293 (±138) % SGA: NR *Mean (±SD) GA* MM: 29.2 (±1.6) Preterm formula: 30.2 (±1)	MM was fortified with bovine-based fortifiers at 100 ml/kg/day There was no difference in volume, energy, or macronutrient intake between groups at hospital discharge Mean LOS was 49.1–52.0 days	−1.1 (±0.7) Mean (±SD) length change from birth to discharge (z-score) −1.0 (±0.6) Mean (±SD) head circumference change from birth to discharge (z score) −1.0 (±0.7) Mean (±SD) body fat mass (g) Discharge: 242 (±99) Term-corrected age (TCA): 458 (±118) Mean (±SD) body fat-free mass (g) Discharge: 1,877 (±371) TCA: 2,622 (±406)	−1.0 (±1.1) Mean (±SD) head circumference change from birth to discharge (z score) −0.9 (±0.8) Mean (±SD) body fat mass (g) Discharge: 297 (±134) TCA: 632 (±141) Mean (±SD) body fat-free mass (g) Discharge: 1,984 (±248) TCA: 2,632 (±249)	compared with the preterm formula group (*p* = 0.018) There were no differences between groups in length or head circumference z-score change. There were also no differences in weight, length, or head circumference at follow-up of TCA, though feeding type in the interim was unclear (data not shown here) Body fat and fat-free mass were not different between groups at discharge. However, by TCA, the preterm formula group had significantly greater fat mass (g and %; *p* = 0.004 and 0.002), and the MM group had significantly greater fat-free mass (% but not g; *p* = 0.002 and NS) Results were not adjusted for confounders	Risk of selection bias
Schanler et al. (35) Prospective cohort study PMID 4032137	*Mean (±SD) birthweight (g)* MM: 1,180 (±35) Formula: 1,195 (±30) All infants were AGA *Mean (±SD) GA* *(weeks):* MM: 29.0 (±0.2) Formula: 29.0 (±0.2)	Infants received MM fortified with skim and cream from donor milk or bovine fortifier. Infants in the formula group received 100 kcal/dl and then 80 kcal/dl formula The intervention continued until infants were 1,800 g, about 8 weeks	Fortified MM (*N* = 14) Mean (±SEM) weight gain (g/kg/day): 22 (±3) Mean (±SEM) length gain (cm/week) (±0.1) Mean (±SEM) head circumference (cm/week) 0.9 (±0.1) Mean (±SEM) sum skinfolds (mm/week) 0.6 (±0.1)	Formula (*N* = 10) Mean (±SEM) weight gain (g/kg/day): 21 (±1) Mean (±SEM) length gain (cm/week) 1.3 (±0.1) Mean (±SEM) head circumference (cm/week) (±0.2) Mean (±SEM) sum skinfolds (mm/week) 0.9 (±0.2)	There were no differences in weight, length, head circumference, or skinfold gains between groups	
Development						
Birch et al. (23) Hoffman, et al. (30) Cohort study, observational analysis of RCT of *n*−3 fatty acids in formulas PMIDs 1399429 (25) 1386065 (23) 8475899 (30)	*Mean birthweight (g)* MM: 1,265 Soy/marine oil: 1,305 Corn oil: 1,324 Soy oil: 1,277 All AGA *Mean GA (weeks)* MM: 30.0 Soy/marine oil: 30.4 Corn oil: 30.5 Soy oil: 30.1	Infants in the human milk group received at least 75% of feedings from MM. No fortification described Infants in the formula groups received either corn and coconut oil (MCT and linoleic acid (18:2 co-6) as EFA) or soy and coconut oil (MCT and 18:2 co-6 and 18:3 co-3) or soy/marine oil (DHA 0.4%) in the formulas Intervention duration: from 10 days postnatal to 57 weeks of PCA (~27 weeks)	MM (*N* =9) Mean (±SD) visual evoked potentials (VEP) (logMAR) 36 weeks of PCA: 0.51 (±0.27) Mean visual evoked potentials (Snellen) 36 weeks of PCA: 20/65 Mean (±SD) forced-choice preferential looking acuities (logMAR) 36 weeks of PCA: 0.73 (±0.7) Mean forced-choice preferential looking acuities Snellen (Snellen) 36 weeks of PCA: 20.11 Mean (±SD) rod electroretinogram function log threshold (scot td-sec) (*N* = 9) 36 weeks of PCA: 0.41 (±0.59) 57 weeks of PCA: −1.39 (±0.16) Mean (±SD) rod electroretinogram function log Vmax 36 weeks of PCA: 1.20 (±0.14) 57 weeks of PCA: 2.12 (±0.15) Mean (±SD) rod electroretinogram function log k (scot td-sec) (*N* = 9) 36 weeks of PCA: 1.25 (±0.54) 57 weeks of PCA: 0.43 (±0.11) Mean (±SD) cone electroretinogram function log threshold (pot td-sec) (*N* = 9)	Formulas: soy/marine oil (*N* = 13) Corn oil (*N* = 12) Soy oil (*N* = 16) Mean (±SD) visual evoked potentials Soy/marine oil 36 weeks of PCA: 0.40 (±0.25) Corn oil 36 weeks of PCA: 0.67 (±0.15) Soy oil 36 weeks of PCA: 0.63 (±0.22) Mean visual evoked potentials (Snellen) Soy/marine 36 weeks of PCA: 20.5 Corn oil 36 weeks of PCA: 20.95 Soy oil 36 weeks of PCA: 20.85 Mean (±SD) forced-choice preferential looking acuities (logMAR) Soy/marine oil 36 weeks of PCA: 0.79 (±0.1) Corn oil 36 weeks of PCA: 0.91 (±0.11) Soy oil 36 weeks of PCA: 0.85 (±0.13)	At 36 weeks, there was significantly higher VEP (logMAR) in the MM group compared with the corn oil and soy oil-based formula groups. FPL acuities were significantly lower in mother's milk group compared with the corn oil-based formula group (*p* < 0.05 for each measurement method). Infants receiving MM had lower Rod thresholds (*p* < 0.05) and log k (*p* < 0.05) than those receiving corn oil-based formula at 36, but not 57 weeks. There were no differences in cone function at either time point.	Risk of selection, attrition, performance, detection bias
				36 weeks of PCA: −0.11 (±0.41) 57 weeks of PCA: −0.20 (±0.27) Mean forced-choice preferential looking acuities Snellen (Snellen) Soy/marine oil 36 weeks of PCA: 20.13 Corn oil 36 weeks of PCA: 20.17 Soy oil 36 weeks of PCA: 20.14 Mean (±SD) cone electroretinogram function CFF (Hz) 0.3 microV criterion 36 weeks of PCA: 51.5 (±6.6) 57 weeks of PCA: 53.1 (±10.0)	Mean (±SD) rod electroretinogram function log threshold (scot td-sec) Soy/Marine Oil (*N* = 14) 36 weeks of PCA: 0.41 (±0.61) 57 weeks of PCA: −1.35 (±0.28) Corn oil (*N* = 12) 36 weeks of PCA: 1.08 (±0.37) 57 weeks of PCA: −1.32 (±0.20) Soy oil (*N* = 17) 36 weeks of PCA: 0.71 (±0.59) 57 weeks of PCA: −1.38 (±0.26) Mean (±SD) rod electroretinogram function log Vmax Soy/marine oil (*N* = 14) 36 weeks of PCA: 1.22 (±0.20) 57 weeks of PCA: 2.09 (±0.18) Corn Oil (*N* = 12) 36 weeks of PCA: 1.05 (±0.11) 57 weeks of PCA: 2.15 (±0.10) Soy oil (*N* = 17) 36 weeks of PCA: 1.08 (±0.20) 57 weeks of PCA: 2.13 (±0.08) Mean (±SD) rod electroretinogram function log k (scot td-sec) Soy/marine oil (*N* = 14)	
				36 weeks of PCA: 1.24 (±0.47) 57 weeks of PCA: 0.43 (±0.18) Corn oil (*N* = 12) 36 weeks of PCA: 1.12 (±0.28) 57 weeks of PCA: 0.53 (±0.22) Soy oil (*N* = 17) 36 weeks of PCA: 1.73 (±0.55) 57 weeks of PCA: 0.44 (±0.26) Mean (±SD) cone electroretinogram function log threshold Soy/marine oil (*N* = 14) 36 weeks of PCA: −0.09 (±0.32) 57 weeks of PCA: −0.22 (±0.19) Corn oil (*N* = 12) 36 weeks of PCA: 0.1 (±0.24) 57 weeks of PCA: −0.21 (±0.27) Soy oil (*N* = 17) 36 weeks of PCA: −0.09 (±0.32) 57 weeks of PCA: −0.21 (±0.17) Mean (±SD) cone electroretinogram function CFF (Hz) 0.3 microV criterion Soy/marine oil (*N* = 14) 36 weeks of PCA: 52.4 (±8.4) 57 weeks of PCA: 51.6 (±6.5)		
Birch et al. (23) NRCT PMID 8455123	*Birthweight: 1,000–1,500 g*	Infants were fed MM or corn oil-based formula Infants received the intervention until 57 weeks of PCA (4 months adjusted age)	MM (N not reported for each group; 30 total) Mean VEP acuity (logMAR) 57 weeks of PCA: 0.46 [20/58] Mean FPL acuity (logMAR) 57 weeks of PCA: 0.76 [20/115]	Corn oil-based formula (N not reported for each group; 30 total) Mean VEP acuity (logMAR) 57 weeks: 0.71 [20/103] Mean FPL acuity (logMAR) 57 weeks: 0.90 [20/159] Corn oil formula (*N* = 12) 36 weeks of PCA: 51.6 (±9.0) 57 weeks of PCA: 56.0 (±6.1) Soy oil formula (*N* = 17) 36 weeks of PCA: 50.1 (±7.0) 57 weeks of PCA: 52.4 (±6.4)	At 57 weeks of PCA, both VEP and FPL acuity were significantly lower in the group receiving MM compared with the group receiving corn oil-based formula (*p* = 0.04 for each measure)	Risk of selection, performance, and detection bias

*AGA, appropriate for gestational age; BPD, bronchopulmonary dysplasia; FPL, forced-choice preferential looking; GA, gestational age; HMF, human milk fortifier; LBW, low birthweight; MM: maternal milk; N, study sample size; NEC, necrotizing enterocolitis; NICU, neonatal intensive care unit; NR, not reported; OR, odds ratio; PMA, post-menstrual age; PCA, post-conceptual age; RCT, randomized controlled trial; ROP, retinopathy of prematurity; SES, socioeconomic status; SGA, small for gestational age; VEP, visual evoked potential; NRCT, non-randomized controlled trial*.

**Table 3:** Summary of findings table.

**Outcomes**	Anticipated absolute effects^*^ **(95% CI)**		**Relative effect (95% CI)**	**No. of participants (studies)**	**Certainty of the evidence (GRADE)**	**Comments**
	**Risk with exclusive formula**	**Risk with ≥75% human milk**				
Mortality Follow-up: range 50 days to 54 days	82 per 1,000	35 per 1,000 (16–75)	OR 0.409 (0.184–0.911)	498 (1 observational study) (31)	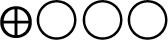 very low^a^	Providing very low birthweight preterm infants with exclusive MM compared with exclusive preterm formula was not associated with incidence of death prior to hospital discharge.
Necrotizing enterocolitis Follow-up: range 6 to 11 weeks	62 per 1,000	35 per 1,000 (14–84)	OR 0.550 (0.22–1.39)	598 (3 observational studies) (29, 31, 34)	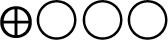 very low^b^	Providing near-exclusive MM to very low birthweight preterm infants, compared with providing exclusive preterm formula, was not significantly associated with odds of acquiring necrotizing enterocolitis within 6–11 weeks.
Sepsis Follow-up: range 7 weeks to 11 weeks	217 per 1,000	169 per 1,000 (111–247)	OR 0.730 (0.45 to 1.18)	532 (2 observational studies) (31, 34)	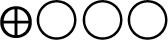 very low^c^	Providing exclusive MM to very-low-birthweight preterm infants, compared with providing exclusive preterm formula, was not significantly associated with odds of sepsis/late-onset sepsis after ~7–11 weeks.
Bronchopulmonary dysplasia Follow-up: mean 11 weeks	261 per 1,000	545 per 1,000 (210–844)	OR 3.400 (0.752 to 15.364)	34 (1 observational study) (34)	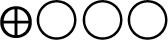 very low^d^^,^^e^	In one small cohort study, providing exclusive MM to very-low-birthweight preterm infants, compared with providing exclusive preterm formula, was not significantly associated with the odds of developing bronchopulmonary dysplasia.
Retinopathy of prematurity Follow-up: range 7 weeks to 11 weeks	116 per 1,000	14 per 1,000 (5–39)	OR 0.110 (0.04–0.31)	532 (2 observational studies) (31, 34)	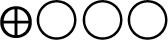 very low^c^^,^^f^	Providing exclusive MM to very-low-birthweight preterm infants, compared with providing exclusive preterm formula, was associated with lower odds of retinopathy of prematurity after 7–11 weeks.
Visual Acuity		Not estimable	–	(2 observational studies) (24, 25, 30)	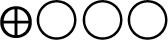 very low^b^^,^^e^^,^^f^	Two studies examined the relationship between providing MM or various formulas that varied by fat sources until up to 57 weeks of PCA in very-low-birthweight preterm infants, and findings were unclear due to inconsistencies between studies and the use of experimental (non-commercially available) formulas.
Weight gain Follow-up: range 2 weeks to 6 months	–	SMD 0.3 SD higher (0.55 lower to 0.06 higher) Fortified groups SMD –0.30 lower (−0.66 to 0.06) Not fortified SMD, −0.57 lower (−1.10 to −0.05)	–	360 (11 observational studies) (21, 22, 26–29, 32–35)	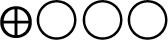 very low ^g^	When very-low-birthweight preterm infants were provided with ≥75% fortified MM, there was no difference in weight gain compared with infants receiving exclusive preterm formula.
Length gain Follow-up: range 2 weeks to 120 days	-	SMD 0.28 SD higher (0.53 lower to 0.05 higher)	–	270 (8 observational studies) (21, 22, 26, 27, 32–35)	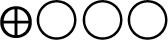 very low ^g^	When very-low-birthweight preterm infants were provided with ≥75% fortified MM, there was no difference in length gain compared with infants receiving exclusive preterm formula.
Head circumference Follow-up: range 2 to 12 weeks	–	SMD 0.25 SD lower (0.53 lower to 0.03 higher)	–	240 (7 observational studies) (21, 22, 27, 32–35)	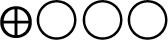 very low ^c^	When very-low-birthweight preterm infants were provided with ≥75% fortified MM, there was no difference in head circumference gain compared with infants receiving exclusive preterm formula.
Fat mass and fat-free mass		Not estimable	–	66 (2 observational studies) (33, 34)	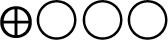 very low^d^^,^^e^^,^^f^	In very-low-birthweight preterm infants, the relationship between providing exclusive fortified MM or preterm formula and body composition was unclear.
Skinfold measures Follow-up: mean 6 weeks		Not estimable	–	24 (1 observational study) (35)	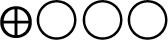 very low^b^^,^^e^	In very-low-birthweight preterm infants, one small cohort study found no relationship between providing fortified MM compared with formula and gains in skinfold measurements after ~8 weeks.

* *The risk in the intervention group (and its 95% CI) was based on the assumed risk in the comparison group and the relative effect of the intervention (and its 95% CI)*.

*CI, Confidence interval; SMD, standardized mean difference*.

*GRADE Working Group grades of evidence*.

*High certainty: We are very confident that the true effect lies close to that of the estimate of the effect*.

*Moderate certainty: We are moderately confident in the effect estimate: the true effect is likely to be close to the estimate of the effect, but there is a possibility that it is substantially different*.

*Low certainty: Our confidence in the effect estimate is limited: the true effect may be substantially different from the estimate of the effect. Very low certainty: We have very little confidence in the effect estimate: the true effect is likely to be substantially different from the estimate of effect*.

a *Risk of selection and attrition bias*.

b *Risk of selection, attrition, performance, detection, and reporting bias*.

c *Risk of selection, attrition, and performance bias*.

d *Inconsistent results between studies*.

e *Risk of selection and performance bias*.

f *Small sample size*.

g *Each study demonstrated risk of selection bias, but attrition, performance, and detection bias were also present throughout the included studies and several studies demonstrated risk of bias in three or four domains. Risk of selection, performance, detection, and reporting bias*.

None of the identified studies controlled for social determinants of health, maternal morbidities, or smoking. Due to the ethical nature of breastfeeding choice, there was no evidence from high-quality randomized studies.

### Mortality

One study demonstrating risk of selection and attrition bias examined the relationship between exclusive fortified MM and exclusive preterm formula and death by time of hospital discharge (31). The authors reported no significant difference in the incidence of death between groups (3.6 vs. 8.2%; *p* = 0.18); however, per analysis by authors of this SR, the results were statistically significant. SR authors attempted to contact study authors for clarification; unfortunately, no response was received. *Conclusion*: In VLBW preterm infants, the relationship between providing exclusive fortified MM or preterm formula and death prior to hospital discharge is uncertain.

*Grade*: very low.

### Necrotizing Enterocolitis

**Figure 2:**
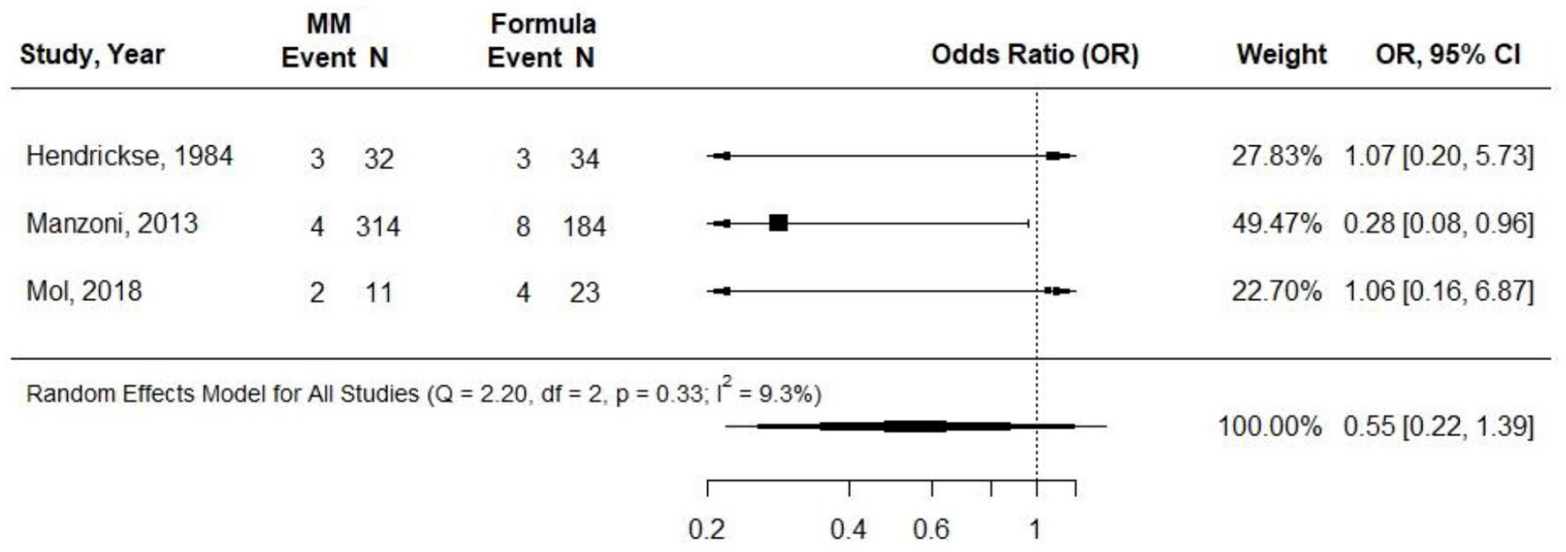
Forest plot demonstrating OR (95%CI) of NEC for VLBW preterm infants receiving ≥75% intake from Maternal Milk (MM) compared to exclusive formula intake.

Three cohort studies examined the relationship between providing VLBW preterm infants with either at least 75% MM or exclusive formula and incidence of NEC (29, 31, 34). In Manzoni et al. (31) and Mol et al. (34), the MM intake was exclusive, and in Hendrickse et al. (29), MM was provided for more than 95% of feedings but was supplemented with SMA Gold cap formula in some cases. MM fortification was not described in Manzoni et al. (31) or Henkdrickse et al. (29) and was described as being at a “standard dose” beginning when milk intake was 140 ml per kg per day in Mol et al. (34). Manzoni et al. was contacted via email, and they confirmed that MM was fortified (31). Infants in the formula group received preterm formula in Manzoni et al. (31) and Mol et al. (34) and LBW formula in Hendrickse et al. (29). Sample sizes ranged from 24 in Hendrickse et al. (29) and 34 in Mol et al. to 498 in Manzoni et al. Observations ranged from 6 to 11 weeks. All included studies demonstrated risk of selection bias. Hendrickse et al. demonstrated risk of selection, attrition, performance, detection, and reporting bias. None of the studies found any difference in odds of NEC when comparing groups receiving near-exclusive MM and exclusive preterm formula. Hendrickse et al. reported that two infants died from NEC (both were receiving exclusive MM) (29). In a meta-analysis of all three studies, there was no statistically significant difference in the OR for NEC according to the infant feeding group (0.55; 95% CI 0.22 to 1.39; *I*^2^ = 9.3%) (Figure 2). *Conclusion*: Very-low-quality evidence suggested that providing near-exclusive MM to VLBW preterm infants, compared with providing exclusive preterm formula, likely results in little to no difference in the odds of acquiring NEC within 6–11 weeks [OR (95% CI): 0.55 (0.22, 1.39)].

*Grade*: very low.

### Sepsis

**Figure 3:**
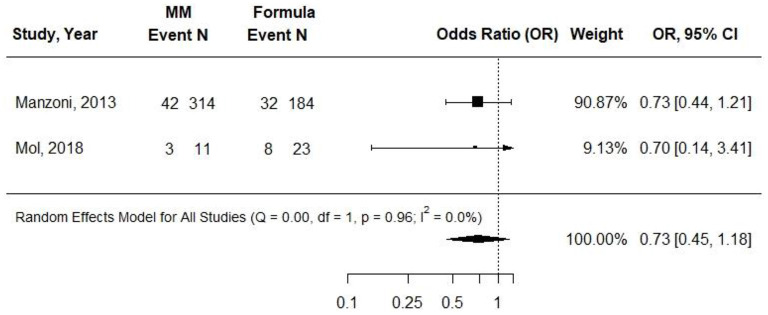
Forest plot demonstrating OR (95%CI) of sepsis for VLBW preterm infants receiving ≥75% intake from MM compared to exclusive formula intake.

Two cohort studies evaluated associations between exclusive MM (≥75%) intake compared with exclusive formula intake and sepsis or late-onset sepsis in VLBW preterm infants (31, 34). MM fortification was not described in Manzoni et al. and was described as being at a “standard dose,” beginning at when milk intake was 140 ml per kg per day in Mol et al. Infants in the formula group received preterm formula in both studies, and observations ranged from 7 to 11 weeks. Both studies demonstrated risk of selection bias: Manzoni et al. demonstrated risk of attrition bias; Mol et al. demonstrated risk of performance bias. Manzoni et al. found no difference in odds of late-onset sepsis between groups, and Mol et al. found no difference in the incidence of sepsis between groups (31, 34). In a pooled analysis, there was no difference in odds of late-onset sepsis or sepsis between groups (OR (95: CI): 0.73 (0.45–1.18); *I*^2^ = 0%) (Figure 3). *Conclusion*: Very-low-quality evidence suggested that providing exclusive MM to VLBW preterm infants, compared with providing exclusive preterm formula, likely results in little to no difference in odds of sepsis/late-onset sepsis after ~7–11 weeks [OR (95% CI): 0.73 (0.45, 1.18)].

*Grade*: very low.

### Bronchopulmonary Dysplasia

One prospective cohort study evaluated associations between exclusive MM (≥75%) intake compared with exclusive formula intake and BPD in VLBW preterm infants (34). Intake of MM resulted in no significant difference in the number of infants who developed BPD by 40 weeks post-menstrual age between groups (54.5 vs. 26.1%, *p* = 0.12; n = 34). *Conclusion*: In one small cohort study, providing ≥75% MM to VLBW preterm infants, compared with providing exclusive preterm formula, likely results in little to no difference in the odds of developing BPD.

*Grade*: very low.

### Retinopathy of Prematurity

**Figure 4:**
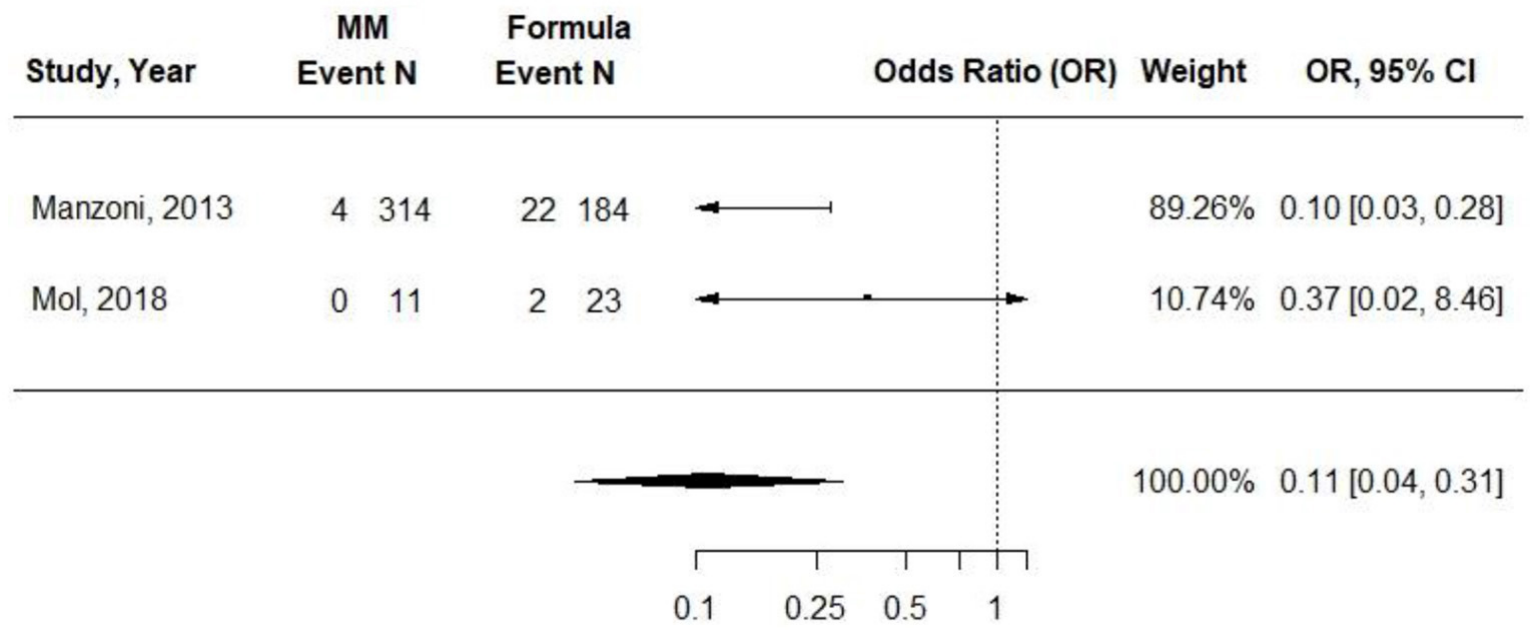
Forest plot demonstrating OR (95%CI) of ROP for VLBW preterm infants receiving ≥75% intake from Maternal Milk (MM) compared to exclusive formula intake.

Two cohort studies evaluated the associations between exclusive MM (≥75%) compared with exclusive formula intake and ROP in VLBW preterm infants (31, 34). Infants in the formula groups received preterm formulas. Sample sizes ranged from 34 to 498, and observations ranged from 7 to 11 weeks. The small study by Mol et al. found no difference in odds of ROP at 40 weeks PMA. In the study by Manzoni et al., infants receiving exclusive MM had an OR of 0.19 (95% CI, 0.05 to 0.69) of threshold ROP compared with those receiving preterm formula (*p* = 0.009) in univariate analysis. The authors did report odds of ROP according to preterm formula feeding, which were adjusted for confounding factors, but there appeared to be an error in the CI, and adjusted data that could be used in a pooled analysis were not included. In a pooled analysis from both studies, there was a significantly decreased OR in ROP for infants fed exclusively with MM compared with infants fed exclusively with preterm formula [0.11 (95% CI) 0.04–0.31; *I*^2^ = 0%] (Figure 4), but the results were not adjusted for potentially confounding variables. *Conclusion*: Providing ≥75% MM to VLBW preterm infants, compared with providing exclusive preterm formula, is associated with lower odds of ROP after 7–11 weeks [OR (95% CI): 0.11 (0.04, 0.31)].

*Grade*: very low.

### Visual Acuity

Two studies by Birch et al. examined the association between infant feeding type (MM without fortification vs. infant formulas) and visual acuity in secondary analyses of randomized trials (23–25, 30). Sample sizes ranged from 52 to 60 participants. These studies demonstrated risk of bias in all domains. Results were not adjusted for confounding variables in either study.

One study by Birch (23, 25, 30) examined the association between infant feeding type (MM without fortification vs. infant formulas containing fats that included soy or marine, corn, or soy oil) and visual acuity. At 36 weeks post-conceptual age (PCA), there was a significantly higher visual evoked potential (VEP) (logMAR) in the MM group compared with the corn oil and soy oil infant formula groups. Forced-choice preferential-looking (FPL) acuities were significantly lower compared with the corn infant formula group (*p* < 0.05 for each measurement method). Infants receiving MM had lower rod thresholds (*p* < 0.05) and log k (*p* < 0.05) than those receiving corn infant formula at 36, but not at 57 weeks. There were no differences in cone function at either time point. In another study by Birch et al. (24), infants were fed MM or corn oil containing infant formula (*n* = 30) until 57 weeks of PCA (4 months of adjusted age). At 57 weeks of PCA, both VEP and FPL acuity were significantly lower in the group receiving MM compared with the group receiving corn oil containing infant formula (*p* = 0.04 for each measure). *Conclusion*: Two studies examined the relationship between providing unfortified MM or infant formulas until up to 57 weeks of PCA in VLBW preterm infants and visual acuity, and the findings were unclear due to inconsistencies between studies and the use of experimental (non-commercially available) formulas.

*Grade*: very low.

### Weight Gain

Ten cohort studies evaluated associations between exclusive MM (≥75%) intake compared with exclusive formula intake and weight gain in VLBW preterm infants (21, 22, 26–29, 32–35). Infants in the MM group received MM exclusively in five studies (26, 28, 33–35) and received ≥75% to <100% in three studies (27, 29, 32). The authors indicated that MM was fortified in five studies (27, 32–35). In most of the studies, the formula consumed in the comparison group was either preterm or LBW formula, though the formula type was not reported in Genzel Boroviczeny et al. Observation duration ranged from 2 weeks to ~3 months (21, 27). Each study demonstrated risk of selection bias, but attrition, performance, and detection bias were also present throughout the included studies, and several studies demonstrated risk of bias in three or four domains. Most of the studies had sample sizes ranging from 10 to 34, but Doege et al. (*n* = 120) and Birch et al. (*n* = 83) had larger sample sizes. None of the studies found that weight gain was greater in the group receiving MM. Half of the studies found no difference in weight gain between groups (21, 22, 27, 28, 32, 35); the other half found that the group receiving MM had significantly less weight gain over the study duration than did the group receiving formula (22, 26, 29, 33, 34).

**Figure 5:**
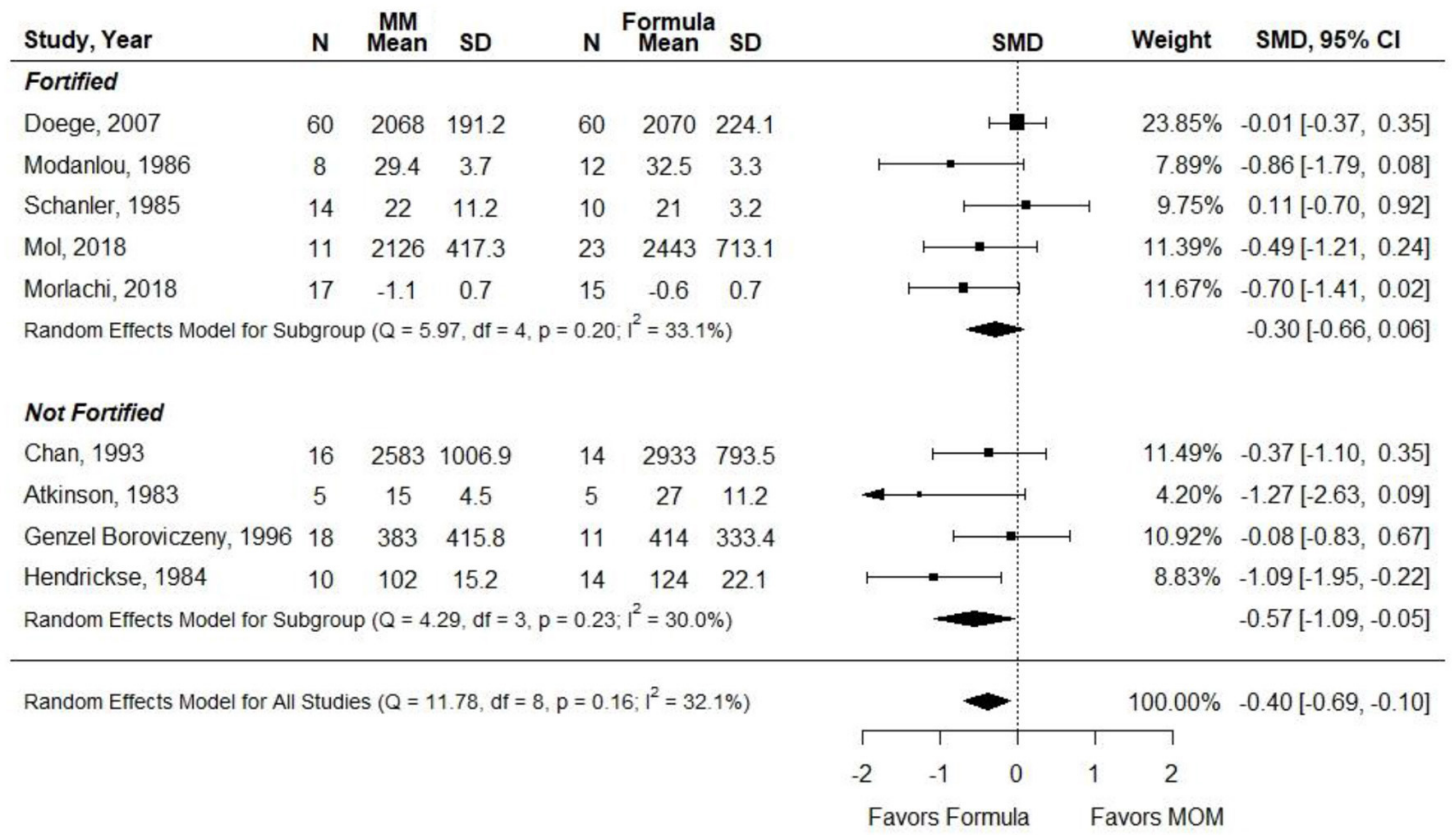
Forest plot demonstrating SMD (95%CI) of weight gain for VLBW preterm infants receiving ≥75% intake from Maternal Milk (MM) compared to exclusive formula intake.

Atkinson et al. did not report data that could be included in a pooled analysis. The measure of SMD was used in a meta-analysis due to heterogeneity in how the outcome was reported (e.g., g, g per day, and g per kg per day). Results were stratified according to whether MM was fortified. When infants in the MM group were given unfortified MM, they had significantly less weight gain than infants receiving preterm or LBW formula (SMD, −0.57; 95% CI, −1.10 to −0.05). However, when MM was fortified, there was no significant difference in weight gain between groups (−0.30; −0.66 to 0.06) (Figure 5). *Conclusion*: When VLBW infants were provided with ≥75% fortified MM, there was no difference in weight gain as compared with infants receiving exclusive preterm formula.

*Grade*: very low.

### Length

Eight cohort studies evaluated associations between exclusive MM (≥75%) intake compared with exclusive formula intake and length gain in VLBW preterm infants (21, 22, 26, 27, 32–35). Infants in the MM groups received MM exclusively in Chan et al., Mol et al., Morlacchi et al., and Schanler et al. and received 75% to <100% in Modanlou et al. and Doege et al. In most of the studies, the formula consumed in the respective group was either preterm or LBW formula. Observation duration ranged from 2 weeks to 120 days (21, 26). Due to the ethical nature of breastfeeding choice, there was no evidence from high-quality randomized studies. Each study demonstrated risk of selection bias, but attrition, performance, and detection bias were also present throughout the included studies, and several studies demonstrated risk of bias in three or four domains. Most of the studies had sample sizes ranging from 10 to 34, but Doege had a sample size of 120. None of the studies found that length gain was greater in the group receiving MM. Six studies found no difference in length gain between groups (21, 27, 32–35). Two studies found that the groups receiving MM had significantly less length gain over the study duration, than had with the groups receiving formula (21, 26).

**Figure 6:**
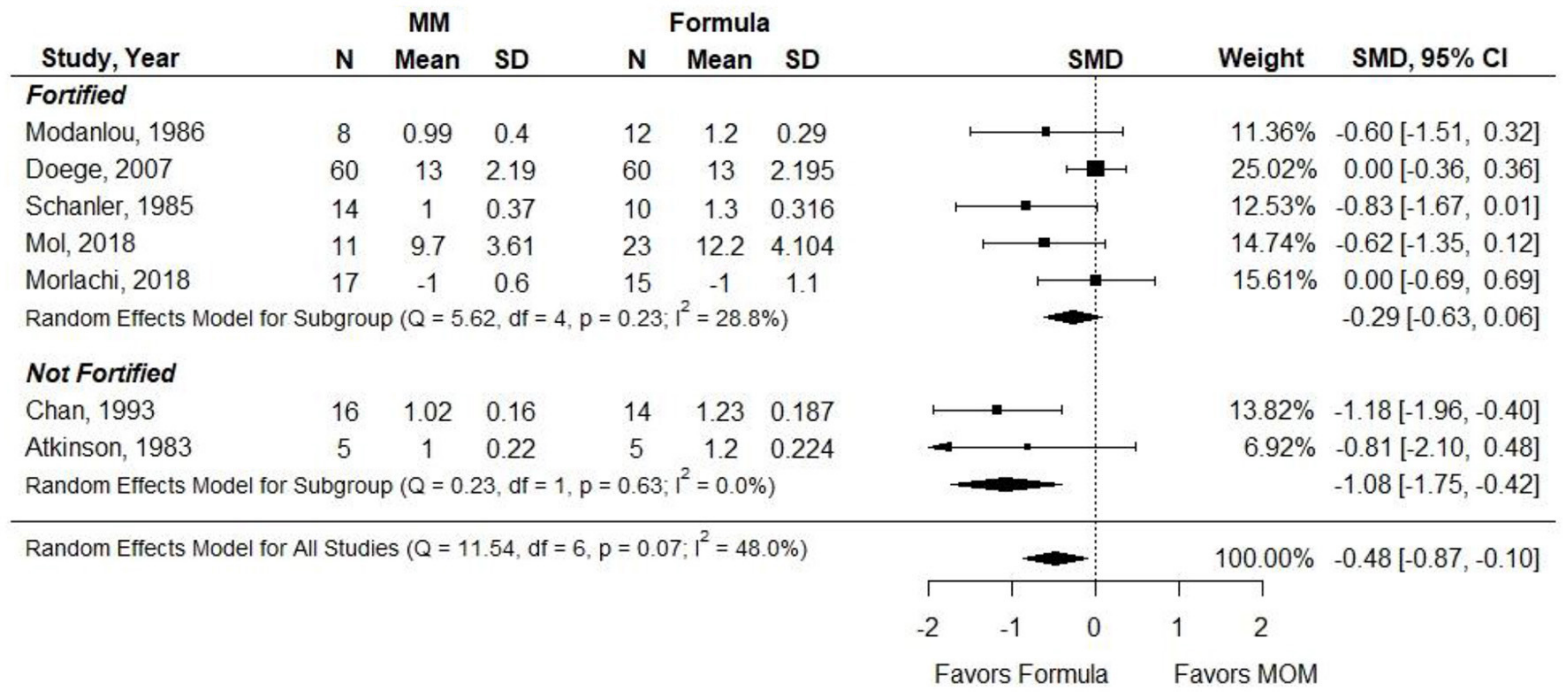
Forest plot demonstrating SMD (95%CI) of length gain for VLBW preterm infants receiving ≥75% intake from Maternal Milk (MM) compared to exclusive formula intake.

Atkinson et al. (21) did not report results that could be included in a meta-analysis. SMD was used as the outcome measure since authors reported length gain using heterogeneous measures (e.g., cm, cm per week, and mm per day). When infants in the MM groups were given unfortified MM, infants in this group had significantly less length gain than infants receiving preterm or LBW formula (SMD, −1.08; 95% CI, −1.75 to −0.42). However, when MM was fortified, there was no significant difference in length gain between groups (−0.28; 95% CI, −0.63 to 0.06) (Figure 6). *Conclusion*: When VLBW preterm infants were provided with ≥75% fortified MM, there was no difference in length gain compared with infants receiving exclusive preterm formula.

*Grade*: very low.

### Head Circumference

Seven cohort studies evaluated associations between exclusive MM (≥75%) compared with exclusive formula intake and head circumference gain in VLBW preterm infants (21, 22, 27, 32–35). Infants in the MM groups received MM exclusively in three studies (33–35) and received 75–100% in two studies (27, 32). The authors indicated that MM was fortified in five studies (27, 32–35). The formula consumed in the respective group was either preterm or LBW formula. Observation duration ranged from 2 to ~12 weeks (21, 27). Each study demonstrated risk of selection bias, but attrition, performance, and detection bias were also present throughout the included studies, and several studies demonstrated risk of bias in three or four domains. Most of the studies had sample sizes ranging from 10 to 34, but Doege had a sample size of 120. None of the studies found that head circumference gain was greater in the group receiving MM. Five studies found no difference in head circumference gain between groups (21, 27, 32–35). Two studies found that the group receiving MM had significantly less head circumference gain over the study duration, compared with the group receiving formula (22, 34).

**Figure 7:**
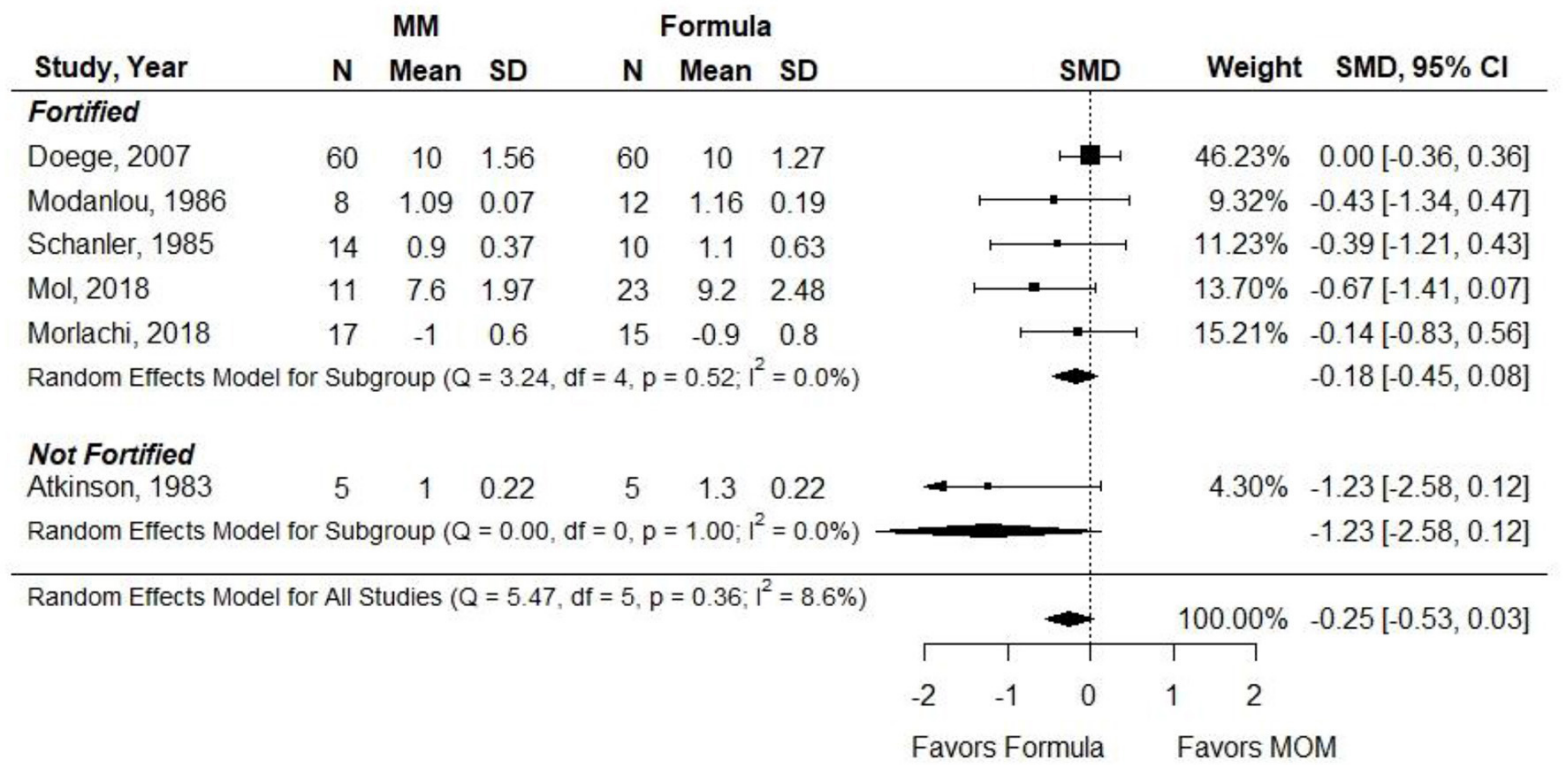
Forest plot demonstrating SMD (95%CI) of Head circumference for VLBW preterm infants receiving ≥75% intake from Maternal Milk (MM) compared to exclusive formula intake.

Atkinson et al. (21) did not report results that could be included in the meta-analysis. SMD was used as the outcome measure since the authors reported head circumference gain using heterogeneous measures (e.g., cm and cm per week). Results were stratified according to whether MM was fortified. There was no difference in head circumference gain between groups [−0.25 (−0.53, 0.03)] (Figure 7). *Conclusion*: When VLBW preterm infants were provided with ≥75% fortified MM, there was no difference in head circumference gain compared with infants receiving exclusive preterm formula.

*Grade*: very low.

### Fat Mass and Fat-Free Mass

Two cohort studies demonstrating risk of selection bias examined the association between providing fortified MM and preterm formula exclusively on fat mass (FM) and fat-free mass (FFM) in VLBW preterm infants (33, 34). Sample sizes ranged from 32 to 34 participants. In Mol et al., there were no differences between groups in FM or FFM at 40 weeks post-menstrual age, although baseline values were not provided. In Morlachi et al., body fat and FFM were not different between groups at discharge. However, by term-corrected age (TCA), the preterm formula group had significantly greater FM (g and percentage; *p* = 0.004 and *p* = 0.002), and the MM group had significantly greater FFM (percentage but not g; *p* = 0.002 and NS). Baseline values were not provided in either study, so the pooled analysis was not possible. *Conclusion*: In VLBW preterm infants, the relationship between providing exclusive fortified MM or preterm formula and body composition is unclear.

*Grade*: very low.

### Skinfold Measurement

One cohort study conducted by Schanler et al. in 1985 demonstrating selection, performance, detection, and reporting bias evaluated the association between providing VLBW preterm infants with MM fortified with either cream from donor milk or bovine fortifier and skinfold measurement (35). Both groups were compared with infants consuming the commercial formula. The formula group included 10 infants, and the MM group fortified with donor milk included 14 infants. Infants in the standard formula group received 100 kcal per dl and then 80 kcal per dl of formula. The intervention continued for about 8 weeks until infants were 1,800 g. There were no differences in skinfold gains between groups.

*Conclusion*: One small cohort study found no relationship between providing fortified MM compared with formula and gains in skinfold measurements after ~8 weeks.

*Grade*: very low.

### Gastrointestinal Health and Bone Mineral Content

No studies were identified that evaluated the association between ≥75% MM intake compared with formula intake and gastrointestinal health or bone mineral content.

## Discussion

The very-low-quality evidence identified through this SR demonstrated that the odds of ROP may be lower with MM intake ≥75% of VLBW preterm infant's enteral nutrition compared with exclusive formula. Specifically, the predominantly MM-fed infants exhibited 0.11 (95% CI 0.04, 0.31) lower odds of ROP after 7–11 weeks (very low quality).

In this meta-analysis of studies specific to VLBW preterm infants, there were no differences observed in mortality, NEC, late-onset sepsis, BPD, or visual acuity according to feeding type (very-low quality evidence for each outcome). The lack of statistical difference between groups in this meta-analysis differs from individual study results, which have shown decreased morbidity, especially in NEC and late-onset sepsis, with the intake of MM (2, 4, 35, 36). These studies did not meet the specific criteria of this SR because they did not compare an exclusively formula-fed group with a predominately MM-fed group and instead compared outcomes based on the dose of MM received. Furthermore, these studies were not limited to preterm infants with birthweight ≤1,500 g. With the known anti-infectious and anti-inflammatory bioactive factors in MM, the potential protection afforded by MM against these infectious and inflammatory diseases is biologically plausible (37–40). The lack of significant difference for these outcomes in this meta-analysis may reflect more the lack of well-designed studies rather than the absence of an effect. Therefore, this meta-analysis serves as a foundation upon which to build future studies to address the role of MM intake in VLBW preterm infant morbidity and mortality.

For anthropometric growth, the results differed by whether or not MM was fortified. There were no differences in weight, length, and head circumference between infants fed fortified MM and those fed LBW or preterm formula. In contrast, when MM was not fortified, the infants' weight gain and length gain were significantly lower than those of the formula-fed infants. There was no difference in head circumference growth, regardless of whether the human milk was fortified. Similar results were found in the SR conducted by Suganuma et al. in 2021, in which there was no significant association between short-term growth outcomes with feeding type (7). The authors reported insufficient evidence to determine effects on any outcomes.

Selection bias was pervasive in the studies included in meta-analyses. Due to maternal autonomy in whether a mother provides her own milk, randomization of infants to MM or formula is not ethical. Since the provision of MM depends on a maternal decision as well as maternal lactation physiology, selection bias is a universal limitation in studies of MM vs. formula feeding. Moreover, at this time, social determinants of health are associated with maternal lactation success (41, 42). None of the studies included in this SR controlled for these factors. Therefore, though some limitations in MM studies are fixed, others are modifiable and should be measured and compared in future studies of VLBW preterm infant outcomes in relation to MM intake. Another bias that is common in studies comparing predominantly MM and formula-fed VLBW preterm infants is performance bias since blinding, like randomization, is difficult in these studies. Detection bias and reporting bias also occurred but not as frequently. These biases are especially hard to avoid in studies comparing the extremes of milk intake, predominately MM and exclusively formula, as the mothers in these two groups may vary greatly in intent and ability to provide milk, morbidities related to lactation insufficiency, maternal self-efficacy regarding milk production, and maternal stress (2, 43–51). Studies comparing less extreme proportions of MM may have fewer inherent differences between groups. Lack of randomized studies and potential for infant health outcomes to be affected by maternal factors may influence MM expression and therefore warrant consideration in the interpretation of these results.

Another limitation of the review was that one of the studies that met the inclusion criteria was a post-discharge study of weight gain (26), and differences between human milk and formula on weight gain may be different after discharge.

Despite these limitations, opportunities to improve the design of infant feeding studies do exist. The collection of data to assess social determinants of health, maternal morbidities related to milk production, and maternal intent for infant feeding would provide opportunities for adjusted models to focus the comparison on the bioactive components of milk rather than the factors related to milk production. Future studies should report the amount of MM intake, donor milk, formula, and any fortification as well as limitations from the observational nature and lack of randomization of these studies. Longer-term studies are needed to further assess morbidities, mortality, and developmental outcomes.

The strengths of this review include its focus on VLBW preterm infants, its broad and comprehensive literature search, the inclusion of the highest level of evidence available, strict inclusion criteria to improve directness and precision of evidence including a focus on MM only, and its recognition of inadequate attention to factors related to MM production such as social determinants of health. The limitations of this SR are reflective of the primary included literature, including risk of selection and other biases that limit the certainty of conclusions.

## Conclusions

This SR demonstrates that fortified MM in comparison with formula may decrease odds of ROP for VLBW preterm infants, but no effect was found in all other outcomes. Given the observational nature of human milk research, cause-and-effect evidence was lacking. Future research should include minimization of bias through careful and standardized measurement of milk intake and important confounding variables, including social determinants of health. The results of this review were utilized in an evidence to decision framework by the Preterm Panel to develop evidence-based VLBW preterm infant enteral feeding recommendations (52).

## Data Availability Statement

The original contributions presented in the study are included in the article, further inquiries can be directed to the corresponding author/s.

## Author Contributions

ST, TF, MR, and LM wrote the first draft of this manuscript. All authors reviewed and commented on subsequent drafts of the manuscript. All authors were involved in developing this systematic review, from question formulation to evidence grading.

## Funding

This work was supported by the Academy of Nutrition and Dietetics.

## Conflict of Interest

The authors declare that the research was conducted in the absence of any commercial or financial relationships that could be construed as a potential conflict of interest.

## Publisher's Note

All claims expressed in this article are solely those of the authors and do not necessarily represent those of their affiliated organizations, or those of the publisher, the editors and the reviewers. Any product that may be evaluated in this article, or claim that may be made by its manufacturer, is not guaranteed or endorsed by the publisher.
